# Stem Cells for Diabetes Complications: A Future Potential Cure

**DOI:** 10.5041/RMMJ.10283

**Published:** 2017-01-30

**Authors:** Mogher Khamaisi, Sarit Ella Balanson

**Affiliations:** 1 Internal Medicine D, Rambam Health Care Campus and Faculty of Medicine, Technion-Israel Institute of Technology, Haifa, Israel; 2 Institute of Endocrinology, Diabetes & Metabolism, Rambam Health Care Campus and Faculty of Medicine, Technion-Israel Institute of Technology, Haifa, Israel

**Keywords:** Diabetes, macrovascular complications, microvascular complications, progenitor cells, stem cells

## Abstract

Long-standing diabetes leads to structural and functional alterations in both the micro- and the macrovasculature. Designing therapies to repair these abnormalities present unique and sophisticated challenges. Vascular endothelial cells are the primary cells damaged by hyperglycemia-induced adverse effects. Vascular stem cells that give rise to endothelial progenitor cells and mesenchymal progenitor cells represent an attractive target for cell therapy for diabetic patients. In this review, we shed light on challenges and recent advances surrounding stem cell therapies for diabetes vascular complications and discuss limitations for their clinical adoption.

## INTRODUCTION

Diabetes is a serious disease that in 2014 plagued 422 million individuals in total worldwide, with one in 11 adults affected.^1^ The disease is characterized by hyperglycemia due to autoimmune destruction of β-cells in the pancreas (type 1) or insulin resistance, usually due to obesity, with decreased pancreatic insulin production and β-cell failure (type 2).^2^ Metabolic disturbances associated with diabetes cause a vast number of complications ranging from cardiovascular and cerebrovascular diseases to neuropathy, retinopathy, nephropathy, and poor wound healing. These complications decrease the quality of life and can lead to death; the most common cause of mortality for diabetic patients is cardiovascular disease.^3^

Current therapies have revolutionized diabetes, no longer making it a death sentence. The therapies target hyperglycemic states, but even when patients have remarkably good glycemic control devastating complications still occur. This is probably because diabetes is a more complicated pathology than we currently understand. The only curative therapy available is pancreatic islet cell replacement, requiring not only rare donors but also immunosuppressant therapy to reduce rejection, which comes with its own complications. Xenografts have been proposed, but the associated complications beg for better alternative sources. New research advocates the use of pluripotent human embryonic stem (hES) cells as a potential new replacement cell therapy for type 1 diabetes mellitus (T1DM). Several investigators have been able to demonstrate the ability to produce hES that function like β-cells: glucose-sensing and insulin-secreting. For example, among the earliest in the field, Assady et al. have been able to use hES cells to produce spontaneous “lineage specific differentiation” of cells that behave and function like β-cells—producing insulin and secreting the substance based on the level of cell differentiation. Their paper proves that hES cells can be easily coaxed into β-cell forms and are a potential source and basis for future cell replacement therapies in diabetes.^4^

Beyond creating new β-cells, current literature supports the use of stem cells to prevent and treat the varied diabetes complications. Two types of multipotent stem cells exist in our bone marrow: mesenchymal stem cells (MSCs) and hematopoietic stem cells (HSCs). Hematopoietic stem cells mature to produce all the cells in the bloodline, while MSCs are involved in tissue regeneration and mature to produce various organs such as bone, cartilage, heart, brain, and kidneys. These stem cells may be utilized as injectable progenitors. Recent research has demonstrated that vascular complications including retinopathy, neuropathy, wound healing, and erectile dysfunction can be targeted via HSCs, whereas renal complications can be targeted via MSCs. Induced pluripotent stem cells (also known as iPS cells or iPSCs) are a type of pluripotent stem cell that can be generated directly from adult cells such as human fibroblasts or human renal epithelial cells. The iPSC technology was first reported by Shinya Yamakana’s laboratory in Japan, showing in 2006 that the introduction of four specific genes encoding transcription factors could convert normal adult cells into pluripotent stem cells. He was awarded the 2012 Nobel Prize along with Sir John Gurdon for the discovery that mature adult human cells can be reprogrammed to become pluripotent.^5^

**Figure 1:**
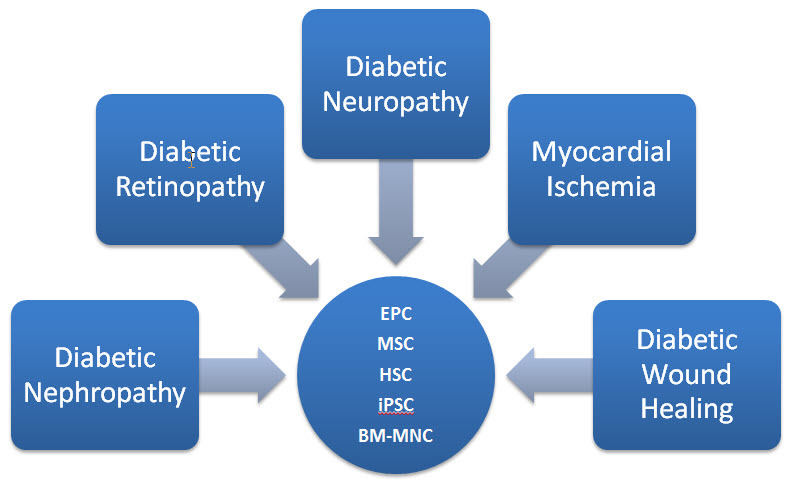
Stem Cell Therapy Options for Treatment of Vascular Complications in Diabetes. BM-MNC, bone marrow derived mononuclear cell; EPC, endothelial progenitor cell; HSC, hematopoietic stem cell; iPSC, induced pluripotent stem cell; MSC, mesenchymal stem cell.

In this report we summarize the current literature about the potential use of stem cells for treatment of micro- and macrovascular complications of diabetes (Figure 1).

## USE OF ENDOTHELIAL PROGENITOR CELLS TO RESOLVE DIABETIC VASCULAR COMPLICATIONS

The range of vascular complications of diabetes is vast, and the exact causes and mechanisms of these complications remain unclear.^6^ However, the latest literature points to endothelial dysfunction as the main initiator. This type of dysfunction is characterized by impaired reaction of the endothelium to stress and ischemia. The complications are associated with dysregulation of vascular remodeling due to pro-inflammatory and proliferative changes that result in increased permeability and decreased responsiveness to noxious stimuli like stress, ischemia, or hypoxia. Further, the injured endothelium does not regenerate, and the increased permeability leads to poor angiogenesis—all of which exacerbates the vascular complications seen in diabetes.

The discovery of endothelial progenitor cells (EPCs) in peripheral human blood and the characterization of EPCs in diabetic patients have helped the scientific community concretize the role of EPCs in endothelial repair. Endothelial progenitor cells have varying proposed functions throughout the body. Relevant here, however, is their high concentration in subjects with coronary artery disease (CAD), whose vessels require more frequent and intense repair. The current literature suggests that they act via paracrine signaling of “… interleukins, growth factors, and chemokines [such as HFG, IL-8, MCP-1, VEGF, G-CSF, and GM-CSF] that are capable of accelerating vascular network formation *in vivo* and enhancing the healing of ischemic ulcers in diabetic mice.”^7^ Endothelial progenitor cells in diabetic patients are both reduced and inherently dysfunctional.^8^ Studies have demonstrated that the number of EPCs decreases both significantly and linearly with the severity of worsening glucose control. The mechanism of this dysfunction is due to weaker signaling from damaged tissue, which cannot recruit sufficient EPCs from the bone marrow. These fewer cells are inherently problematic, as they cannot release the essential pro-angiogenic and inflammatory factors necessary to elicit good vascular repair. Further, EPCs of diabetic individuals have “decreased migratory prowess and reduced proliferative capacity and an altered cytokine/ growth factor secretory profile that can accelerate deleterious repair mechanisms rather than support proper vascular repair.”^7^ On a paracrine level, diabetic cells release anti-angiogenic hormones, there-by creating an unfavorable environment in which EPCs must function and limiting their ability to promote repair and productive neovascularization.

The thought is that diabetic individuals with vascular complications may benefit from autologous stem cell transplant therapy if *ex vivo* modification of EPCs can be effectively performed. This would provide better repair mechanisms to the damaged vasculature. A number of studies in mice have shown that diabetic EPCs in healthy mice reduce healthy vascularization. The reverse may be true, but few studies directly address this idea in diabetic cells or models.^7^

## DIABETIC NEPHROPATHY

### Mesenchymal Stem Cells for Diabetic Nephropathy

Diabetic nephropathy is the main complication in T1DM. Research has demonstrated that, *in vivo*, MSCs differentiate into renal cells and may repair a damaged segment of a kidney. They have also been shown by Lee et al. to function as reno- and pancreato-protective: in their study MSCs aggregated and prevented further damage to the tissue.^9^ Further, the cells are posited to be “a perfect tool for transplantation” because they are hypoimmunogenic. Ezquer et al. demonstrated in their study that injection of MSCs into diabetic mice reduced hyperglycemia and glycosuria as well as expanded morphologically normal β-cell islets.^10^ Further, albuminuria was reduced, and glomeruli were found to be histologically normal as compared to untreated mice, whose glomeruli showed histologic evidence of disease including hyalinosis and mesangial expansion.^10^ These findings suggest that MSC transplantation therapy may allow for better glycemic control and prevent the development of nephropathy. These results do *not* suggest a mechanism by which the damage is reversed. However, the study does offer a few possibilities: the MSCs might have replaced the damaged renal cells; MSCs might scavenge cytotoxic molecules; MSCs might modulate the inflammatory process; or MSCs might promote neovascularization thereby preventing the nephropathy altogether.^10^

### Potential iPSC Use for Diabetic Nephropathy

Other reports advocating the renal progenitor iPSC (including Lam et al. and Toyohara et al.) have demonstrated that iPSCs can easily and quickly be induced into renal cells and that these iPS renal cells have therapeutic significance because they improve acute kidney injury (AKI) in mouse models.^11^,^12^

Lam et al. efficiently, and, more importantly, reproducibly, generated kidney lineage cells from hES and iPSCs. First, tubule-like structures were generated by placing them in an environment that expressed intermediate mesoderm markers. With further coaxing using fibroblast growth factor-2 and retinoic acid, these structures began expressing proximal tubule markers. Finally, the cells further differentiated and matured in an environment of fibroblast growth factor-9 and activin until they expressed mesenchyme nephron progenitor cell markers. Importantly, the mesodermal cells spontaneously formed the ciliated tubules that expressed the markers found in mesenchyme nephron progenitor cells. These findings are promising for potential use of bioengineered stem cells in transplantation to alleviate some of the burden of AKI, chronic kidney disease (CKD), and end-stage renal disease (ESRD).^11^

A recent study by Toyohara et al. demonstrated the ability to use iPS renal progenitor cells to improve AKI. They were able to use iPSCs to create renal progenitor cells via a differentiation protocol that employed signaling with growth factor signaling molecules like bone morphogenic peptide (BMP) inhibitors and transforming growth factor beta (TGFβ) isoforms to form tubule-like structures *in vitro* and *in vivo*. Further, renal subcapsular transplantation of these iPSCs was able partially to rescue renal injury, measured via urea and creatinine, in mouse models with AKI. These results en-courage the further examination and exploration of iPS renal cells to be used as future regenerative therapies.^12^

## DIABETIC NEUROPATHY

Often neuropathy is one of the first noticeable complications in diabetes as 20%–30% of patients are symptomatic. The pathology of diabetic neuropathy is due to metabolic and vascular disturbances in the vasa nervorum, the blood vessels that supply small peripheral nerves.^13^ As a result, there is nervous cell degeneration resulting in the classic symptoms of neuropathic pain and paresthesia, pre-disposing to infection as the diabetic patient does not feel the small cut on his foot. While rigorous glycemic control does slow progression, it cannot fully prevent the development of polyneuropathy.^14^ Thus, new therapies are desired.

Studies have demonstrated that inducing angiogenesis via secretion of cytokines like vascular endothelial growth factor (VEGF) and basic fibro-blast growth factor (bFGF) ameliorates neuropathic pain by increasing blood supply to the nerves. Below we review studies that demonstrate the use of EPCs, bone marrow mononuclear cells (BM-MNCs), and MSCs for treatment.

### Endothelial Progenitor Cells for Diabetic Neuropathy

Previous studies have shown that pharmacologic methods of increasing blood flow to the vasa nervorum vessels, such as anticoagulation and vasodilation, improve neuropathic symptoms.^15^ Further, in ischemic events, EPCs are released from the bone marrow and home to the site of hypoxia. There, the EPCs differentiate and induce local angiogenesis to repair and revascularize the ischemic tissue.

A study by Naruse et al. showed that EPCs derived from human umbilical cord blood reversed diabetic neuropathy in diabetic rats via neovascularization to rescue dying nerves.^16^ Histological studies demonstrated increased microvessels in the EPC-injected side of rats. The study was able to use each rat as a control by injecting EPCs unilaterally instead of bilaterally. However, the EPCs did not have any effect on the vasculature of healthy rats—perhaps because there was no local VEGF secreted in healthy rats, as there was no ischemic tissue that needed to be revitalized. These results support the use of EPC-induced neovascularization to treat diabetic neuropathy.^16^

### Bone Marrow Mononuclear Cells for Diabetic Neuropathy

As mentioned above, diabetic neuropathy has varied metabolic and vascular causes. On a nerve-level the pathologies include cutaneous endothelium-related vasodilation, C-fiber-mediated vasoconstriction, and epineural blood flow in the sural nerve. Previous animal model studies have demonstrated relief of neuropathic pain using antioxidants like a-lipoic acid, taurine, poly-ADP-ribose polymerase inhibitors, and aldose reductase inhibitors. Further, treatment with C-peptide not only improved neuropathic symptoms but also reduced neurotropic proteins in peripheral nerves.^17^ Regardless, these treatments do not target the underlying pathology, thereby demanding improved therapies.

Studies in the past have shown that BM-MNC transplantation improves post-ischemic neovascularization and causes increased blood flow. The leading theory is that the enhancement is due to secretion of pro-angiogenic ligands and cytokines such as fibroblast growth factor (FGF), VEGF, interleukin 1b (IL-1b), and tumor necrosis factor alpha (TNFα). A study by Naruse et al. demonstrated that BM-MNC transplantation alleviates neuropathic pain in diabetic rats as well as increases blood flow to peripheral nerves. This is observed via increased capillary density, with increased nerve conduction velocity. Thus this proposed therapy of BM-MNC transplantation would target not only the debilitating symptom of neuropathic pain but also the underlying cause, improving nerve function altogether.^17^

### Mesenchymal Stem Cells for Diabetic Neuropathy

Shibata et al. transplanted MSCs in diabetic rats and assessed cell homing as well as the effects of the MSCs on diabetic neuropathy. They discovered that the cells settled in the gaps between muscle fibers but were not incorporated into any new structures. The injected muscles had higher levels of VEGF and bFGF mRNA expression, indicating neovascularization and angiogenesis supporting the regeneration of nerve cells. Further, the MSC-transplanted diabetic rats demonstrated improved pain, increased nerve conduction velocity, increased blood flow, and increased capillary density in the MSC-treated limbs. Secretion of VEGF and bFGF was maintained up to 4 weeks after transplantation.^18^ These findings suggest that MSC transplantation can ameliorate diabetic neuropathic pain and induce angiogenesis via increased pro-angiogenic cytokine production.^19^

## DIABETIC RETINOPATHY

### Use of Stem Cells for Diabetic Retinopathy

Diabetic retinopathy is the main cause of blindness in the developed world.^20^ Like other diabetes complications, strict glucose control helps but does not reverse the progression of the retinopathy. The retinopathy pathology is caused by disruptions in the eye’s vasculature due to the various metabolic disturbances in diabetes. These metabolic disturbances range from VEGF levels to accumulation of glycosylation end products. Current therapies include laser photocoagulation and vitreotomy, but those treatments are neither curative nor do they target the pathologic mechanism of the disease.^21^

Various trials have investigated these ideas in diabetic rat and human models. Using immunohistochemistry, studies were able to demonstrate that intravitreally injected stem cells aggregate and home in on the inner retina; this may improve visual function.^20^ Current human clinical trials are ongoing to evaluate the use, efficacy, and safety of HSC injections to treat retinal vascular diseases in multiple studies by Siqueria et al.^22^ Two patients with diabetic retinopathy were injected with HSCs, and the results have demonstrated improved visual acuity and improved ophthalmic metrics up to 12 weeks after treatment. The mechanism by which the HSCs act is unclear but is perhaps due to paracrine signaling. Animal models have demonstrated that HSCs injected intravitreally rescue retinal damage from light, ischemia, and diabetes.^22^ These few successful studies are incredibly encouraging and urge us to continue with larger human trials. Besides HSCs, other stem cells have been investigated for use in treating diabetic retinopathy, e.g. cells such as MSCs, EPCs, and adipose stromal cells. The various cell types behave similarly via paracrine signaling that induces tissue preservation and often reconstruction of the damaged material.^23^ Specifically, HSCs are recruited to the damaged site to promote healing, in a way similar to their behavior in other diabetic complications.^24^ Adipose stromal cells decrease vascular leakage and apoptosis around vessels, perhaps via pericytes that create tight junctions in blood vessels.^25^ While the studies are small, the therapies employed indicate the potential to reverse damage to retinal tissue by partially rescuing the diabetic retinopathy.

## MYOCARDIAL ISCHEMIA

### Use of Stem Cells for Myocardial Ischemia

Ischemic heart disease is the leading cause of death in the United States. In diabetic patients, cardiovascular complications are the most common cause of death. Current therapies for ischemic heart disease include pharmacologic agents that promote perfusion and/or surgeries to revascularize ischemic areas of the heart. None of these is curative, nor together can they fully reverse the damage—hearts, once injured never work the same as they did before. Thus, the studies discussed in the following section have broader ramifications to both diabetic and non-diabetic patients alike.^26^,^27^

A large randomized trial of POSEIDON patients demonstrated that transendocardial injection of MSCs improves heart remodeling in patients with chronic ischemic cardiomyopathy by reducing scar size and improving ventricular functional responses.^26^ Patients’ scars were assessed using multidetector computed tomography (MDCT), and myocardium was analyzed for segmental early enhancement defect (SEED), segmental ejection fraction (SEF), wall-thickening, and topographic analysis. Results showed that scars were reduced significantly by 43.7% with MSC injection versus 25.1% in non-injected tissue. Ventricular function in injected segments improved significantly by 19.9%–26.3% versus 12.1%–19.9% in non-injected issue.^26^

The investigators also assessed whether sites of injection of autologous MSCs responded more favorably than other parts of the infarcted tissue. The study concluded that the site of injection of MSCs is important: the response at that point showed (1) scar reduction, (2) improvement in wall thickening, and (3) functional recovery. Segmental ejection fraction was only improved at the site of injection, not distal to it. Previous studies have demonstrated that MSCs home to and differentiate in areas of infarct borders and release paracrine mediators that effect scar reduction, leading to increased transmural functionality. This study confirmed what previous studies have shown, but Suncion et al. were also able to capture improvements in cardiac function metrics such as contractility, lusitropy, and myocardial energetics. Cell dosage of MSCs was also assessed to determine best results with the smaller dose, 20 million cells versus 100 million cells, producing a greater reduction in scar size. Further, exact administration of MSCs with respect to location, dosage, and rate should be customized based on the individual’s infarct details.^26^

Another important paper, by Karantalis et al. on the PROMETHEUS trial, assessed MSC transplantation as an adjuvant treatment for those under-going coronary artery bypass grafting.^27^ In the study, six patients undergoing coronary bypass surgery received autologous MSC injections in regions of the heart that were hypokinetic but not receiving bypass graft blood. Magnetic resonance imaging (MRI) assessed structural and functional repair in both MSC-injected sites and bypass graft sites. The result was improved overall left ventricular function in regions treated with MSC injections—measured by end-diastolic volume (EDV), end-systolic volume (ESV), left ventricular stroke volume (LVSV), scar segment size, and wall thickness. The study proposed three mechanisms underlying the reparative response by MSCs: (1) reduced fibrosis, (2) neovascularization, and (3) neomyogenesis. These results suggest that MSCs act primarily locally by significantly decreasing scar tissue, increasing viable tissue, and increasing regional contractility.^27^

## DIABETIC WOUNDS

### Use of Stem Cells for Treatment of Diabetic Wounds

The skin’s healing capacity in patients with diabetes is markedly diminished, resulting in a high risk for chronic wounds.^28^ Stem cell-based therapies have the potential to enhance cutaneous regeneration largely through trophic and paracrine activity. Candidate cell populations for therapeutic application include adult MSCs, ESCs, and iPSCs. Despite current treatment modalities, non-healing diabetic ulcers often result in limb amputations. Patients that have diabetic ulcer complications tend to be sicker and less well controlled. Diabetic populations of mice and rabbits with foot ulcers had reduced levels of EPCs and increased inflammatory cytokines such as C-reactive protein (CRP), IL-8, and TNFα. The increased inflammatory markers correlated to poor and incomplete wound healing.^29^ The literature has theorized that, perhaps, if we can somehow alter these fibroblast characteristics we can promote wound healing. Gerami-Naini et al. were able to generate iPSCs from fibroblasts taken from diabetic patients with diabetic ulcers and reprogram them into fibroblasts. Remarkably, these newly induced fibroblasts from iPSCs from diabetic fibroblasts were similar to those from non-diabetic fibroblasts.^30^ This provides evidence for the potential use of iPSCs to improve wound healing in diabetic patients. Indeed, a study by Khamaisi et al. demonstrated that iPSCs in diabetic models exhibited more efficient wound healing and secreted higher levels of pro-angiogenic factors. The study compared fibroblasts from diabetic patients to iPSCs, with the iPSCs having better promotion of wound healing. Further, the study was able to show that inhibiting protein kinase C delta (PKCδ), an isoform of protein kinase C involved in fibroblast functioning, improves diabetic fibroblast function to that of normal fibroblast function.^31^ Other studies have demonstrated that MSCs can assist in better wound healing as they promote cell migration and neovascularization via paracrine signaling. They also decrease inflammation and scarring. These mechanisms apply not only to diabetic wounds but also to all wounds, especially those of elderly or obese patients.^32^–^34^

## CONCLUSIONS

**Figure 2:**
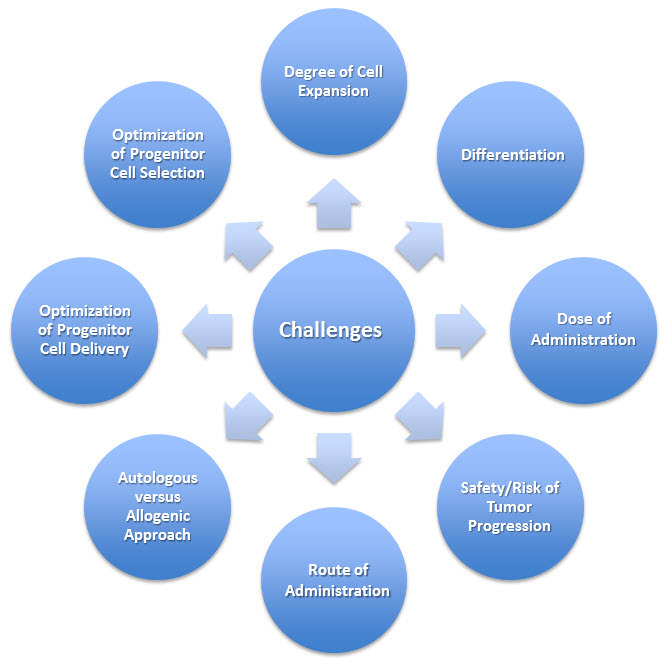
Challenges for the Clinical Application of Stem Cells to Treat Diabetic Vascular Complications.

Microvascular, macrovascular, and inflammatory abnormalities in tissues of patients with diabetes present particular hindrances to adequate treatment of the late disease complications. Stem cells offer a promising approach to these complications in the diabetic setting through release of soluble growth factors and cytokines that stimulate new vessel formation and modulate inflammation. Furthermore, autologous cell-based approaches are ideal to minimize immune rejection but may be limited by the declining cellular function associated with diabetes. However, significant hurdles remain in optimizing progenitor cell selection and delivery (Figure 2). As our understanding of stem cell biology and heterogeneity grows, improved cell selection should lead to observable benefits. Emerging techniques such as micro-fluidic single-cell characterization offer particularly attractive strategies for identifying and isolating the most effective cells for therapeutic use. Despite the multiple barriers to clinical implementation, stem cells have shown sufficient promise to garner a place in the field of regenerative medicine. Progenitor cell therapies have significant implications for human health. More clinical studies are needed to build on recent successes in basic and translational research in hopes of revolutionizing diabetes care in the twenty-first century.

## Abbreviations

AKI: acute kidney injury
bFGF: basic fibroblast growth factor
BM-MNCs: bone marrow mononuclear cells
BMP: bone morphogenic peptide
CRP: C-reactive protein
EC: endothelial cell
EPCs: endothelial progenitor cells
FGF: fibroblast growth factor
hES: human embryonic stem
HSC: hematopoietic stem cell
IL-1b: interleukin 1b
iPSC: induced pluripotent stem cell
MSC: mesenchymal stem cell
PKCδ: protein kinase C delta
T1DM: type 1 diabetes mellitus
TGFβ: transforming growth factor beta
TNFα: tumor necrosis factor alpha
VEGF: vascular endothelial growth factor
